# First case report of human infection with *Mycobacterium stomatepiae*

**DOI:** 10.1099/jmmcr.0.005146

**Published:** 2018-03-22

**Authors:** Jared Weston, Sushil Pandey, Evan Matthews, Evan Bursle

**Affiliations:** ^1^​ Rockhampton Base Hospital, The Range, Rockhampton, Queensland, Australia; ^2^​ Pathology Queensland Mycobacterial Reference Laboratory, Herston, Brisbane, Australia; ^3^​ Rockhampton Mater Hospital, Ward Street, Rockhampton, Queensland, Australia; ^4^​ Central Queensland University, Rockhampton, Queensland, Australia; ^5^​ University of Queensland, Brisbane, Queensland, Australia; ^6^​ Sullivan Nicolaides Pathology, Bowen Hills, Brisbane, Queensland, Australia; ^7^​ Princess Alexandra Hospital, Ipswich Road, Woolloongabba, Queensland, Australia

**Keywords:** nontuberculous Mycobacteria, lymphadenitis, mycobacterial cervical lymphadenitis, scrofula

## Abstract

**Introduction:**

We describe the first detailed case report of human infection with *Mycobacterium stomatepiae*. Infection with non-tuberculous mycobacteria (NTM) related to *M. stomatepiae* is well described, despite the lack of previous confirmed reports of *M. stomatepiae-*related human disease. Localised cervical lymphadenitis is the most common NTM disease in children, with species closely related to *M. stomatepiae,* such as *Mycobacterium triplex* and *Mycobacterium florentinum*, having been shown to be rare causative agents.

**Case presentation:**

A 19-month-old girl presented with persistent unilateral neck lumps which developed following a facial laceration. Both lumps were fluctuant with overlying erythema and no fistulae present. Incision and drainage with curettage was performed. The operative sample of purulent fluid revealed pleomorphic bacilli on Ziehl–Neelsen staining. The isolate cultured was referred for further genotypic identification via 16S rRNA gene sequencing, identifying the organism as *M. stomatepiae*.

**Conclusion:**

We describe the first detailed case report of human infection with *M. stomatepiae.* This organism can now be added to the growing list of NTM that are opportunistic human pathogens, though it is likely to remain a very rare causative agent of this clinical syndrome. Early diagnosis relies on clinical suspicion by the treating doctor, flagging potential cases to the microbiology laboratory and hence allowing correct specimen set-up. Laboratory diagnosis requires incubation of cultures at lower temperatures, and definitive identification is best performed by sequencing methods, including 16S rRNA gene sequencing. The description of novel species of NTM causing human disease is likely to increase with further advancements in diagnostic methods.

## Introduction

We describe the first case of confirmed human infection with *Mycobacterium stomatepiae*. This species can cause infection in some species of fish [1], while other closely related Mycobacterial species have been implicated as rare causes of human infection [2]. Our report adds *Mycobacterium stomatepiae* to the list of potential human pathogens and, possibly, zoonoses. In this case, the exact source of infection could not be determined, but is presumed to be secondary to aquatic environmental exposure. As this species is likely to remain a very rare cause of human disease, the detail regarding diagnosis, treatment and outcome from this report can be used to inform patient management decisions in future cases.

## Case report

A 19-month-old girl with no known immunocompromise presented with persistent unilateral neck lumps two weeks after lacerating her chin on a wooden shelving unit. While the laceration and swelling had healed uneventfully without specific therapy, neck swelling and overlying erythema developed following her injury, further increasing over the following four weeks. An ultrasound revealed several mild to moderately enlarged left submandibular and upper cervical lymph nodes—changes in keeping with inflammation.

**Fig. 1.**
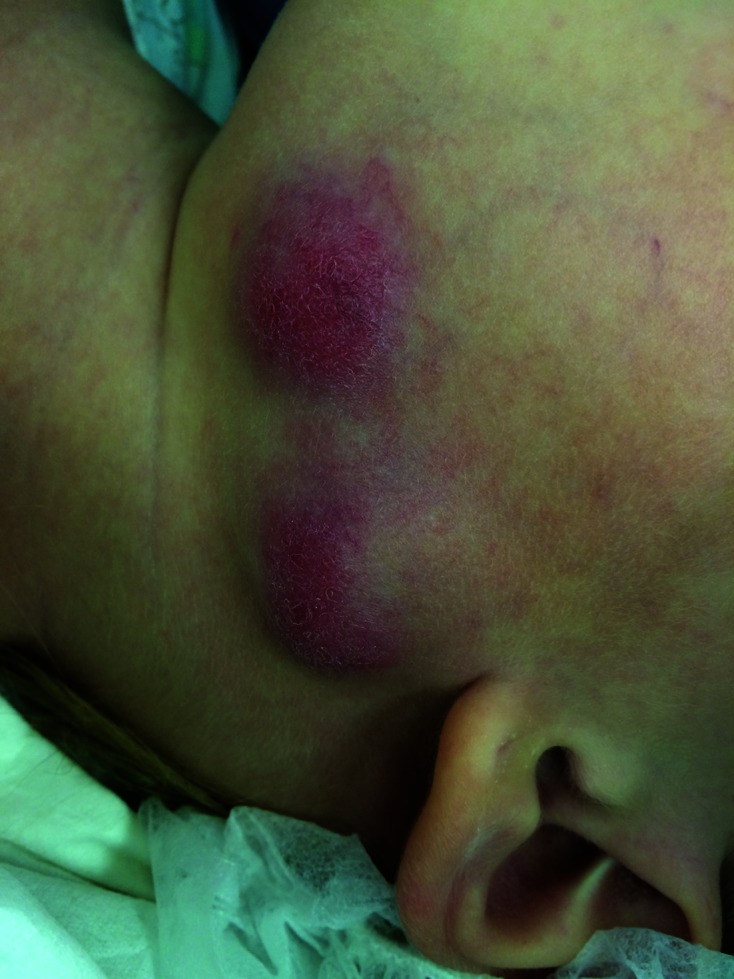
Examination finding of two prominent lymph nodes on the left side of her neck with overlying erythema.

Examination revealed two prominent lymph nodes on the left side of her neck but was otherwise unremarkable. An 18 mm lymph node was located in the submandibular area, and another node (22 mm) towards the tail of her parotid (Fig. 1). Both were fluctuant with erythema of the overlying and surrounding skin. There were no fistulae present. Incision and drainage with curettage was performed under general anaesthesia. Copious purulent discharge was drained and sent along with tissue for histological review and culture. A full blood count performed was within normal limits and C-reactive protein was not elevated. A ten-day course of Clarithromycin 150 mg twice daily was taken post-operatively. The wound healed without complication and no further therapy was required.

## Investigations

**Fig. 2.**
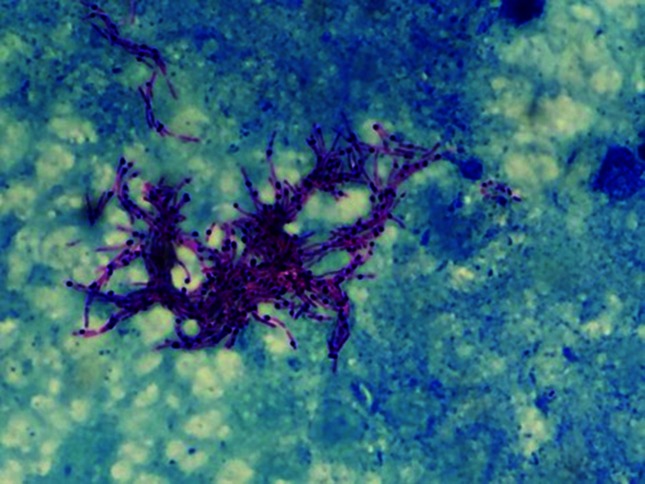
Ziehl–Neelsen stain of the purulent soft tissue specimen revealing pleomorphic bacilli.

The operative sample of purulent fluid was Ziehl–Neelsen (ZN)-stain-positive, with three acid-fast bacilli seen per high powered field. A Gram stain was negative. Standard bacterial and fungal cultures were negative. Bacterial growth was detected after 15 days incubation at 32 °C on Middlebrook 7H11 and chocolate solid media. Media incubated at 36 °C were negative. ZN staining revealed pleomorphic bacilli (Fig. 2). The isolates were referred for further genotypic identification via 16S rRNA sequencing. No resistance testing was performed on the isolate.

### 16S rRNA gene sequencing

The patient isolate was subjected to DNA extraction using a Roche pure PCR template preparation Kit (Roche diagnostics) as per the manufacturer's instructions. Partial 16S rRNA gene PCR amplification with primers BF 5′-GTA AAA CGA CGG CCA GTA GAG TTG GAT CC TGG CTC AG-3′ and R2 5′-CCT ACG AGC TCT TTA CG-3 was performed. Each 50 µl PCR mixture contained 1.0 µl template DNA, 2.5 U *Taq* DNA polymerase, 0.25 mM deoxynucleotide triphosphates, 1.5 mM MgCl_2_ and each primer at a concentration of 0.2 µM. The PCR cycling parameters were 95 °C for 5 min, followed by 35 cycles of 95 °C for 1 min, 55 °C for 1 min and 72 °C for 1 min with a final extension step at 72 °C for 5 min. PCR products were purified with a High Pure PCR purification Kit (Roche Diagnostics) and sequenced using the Big Dye Terminator Cycle sequencing kit with AmpliTaq DNA polymerase (Applied Biosystems) in an ABI Prism 3100 DNA sequencer (Applied Biosystems). The sequences were compared with 16S rRNA gene sequences in the GenBank database by multiple-sequence alignment with clustal W (Ref- https://blast.ncbi.nlm.nih.gov/Blast.cgi). A total of 466 nucleotide positions were included in the analysis. There was a 100 % match between the amplified sequence of the patient isolate and a partial sequence of the 16S rRNA gene of *M. stomatepiae* reference strain DSM 45059^T^ (GenBank accession no. HM022202.1). On the basis of the results of 16S rRNA gene sequencing, this isolate was identified as *M. stomatepiae*.

## Treatment

The lesion was incised and drained and a ten day course of clarithromycin 150 mg twice daily was given.

## Outcome and follow-up

The lesion completely resolved following therapy, with no further treatment required. The patient remained well with no clinical relapse at 3 months follow up.

## Discussion

Here we describe the first detailed case report of human infection with *Mycobacterium stomatepiae. M. stomatepiae* is a slow-growing mycobacteria, closely related to *Mycobacterium florentinum* and part of the *Mycobacterium simiae* complex [1]. The species was first described in 2007 after being identified as the causative agent of fish mycobacteriosis in striped Barombi Mba cichlids (*Stomatepia mariae*) from London Zoo [3]. It has also been isolated from lesions in Kafue leche, a semi-aquatic antelope from south-central Africa [4]. Although the extent of its environmental niche is unknown, these reports indicate that *M. stomatepiae,* like many other non-tuberculous mycobacteria, is likely to have an aquatic predisposition. While *M. stomatepiae* has not been previously described as a human pathogen [1], an isolation of *M. stomatepiae* has been documented [2] in a 26 year old male patient with a cervical abscess. However, no further clinical or exposure information is available regarding this potential case. In our case, a thorough review of all marine exposures in the preceding months was undertaken. The patient had exposure to estuarine and freshwater rivers throughout Central Queensland during recreational activities. No direct contact with marine life was recalled, although the child’s immediate family members frequently fish and subsequent vicarious exposure to diseased fish could not be excluded.

Suppurative cervical lymphadenitis in children is most commonly caused by pathogens such as *Staphylococcus aureus* and *Streptococcus pyogenes*. However, non-tuberculous mycobacteria (NTM) related cervical lymphadenitis is well described (and, in fact, the commonest cause of NTM disease in children), with *Mycobacterium avium* complex species representing the most frequent pathogens. Other mycobacterial species are much less common in this setting, though species related to *M. stomatepiae* (e.g. *M. triplex* and *M. florentinum)* have been shown to be rare causative agents [5].

The description of novel species of NTM causing human disease is likely to increase with the further advancement of diagnostic methods. The expanded use of molecular techniques, particularly nucleic acid sequencing, has allowed vastly improved discrimination of species compared with outdated phenotypic methods [1]. Prior to the use of these technologies, uncommon species such as *M. stomatepiae* may have failed to be identified to species level, or been incorrectly identified as other members of the NTM family. Another benefit of these diagnostic advancements is improved sensitivity. For a fastidious, slow-growing organism such as *M. stomatepiae*, a yield from culture requires lengthy incubation on dedicated media, at a variety of temperatures. Such specialist culture methods may not be requested by clinicians and even if they are, many laboratories lack the capacity to perform it. The subsequent referral to a speciality laboratory for culture has the potential to decrease yield again due to delays in processing. These issues mean such organisms can be easily missed. The increasing application of nucleic acid technologies directly on tissue, however, presents an opportunity to gain an early diagnosis without the constraints of mycobacterial culture. Successful identification of *M. stomatepiae* via direct nucleic acid methods has even been described on formalin-fixed, paraffin-embedded tissue samples [6]. Increasing use of such technology on clinical samples is likely to further increase the identification of unusual NTM, such as *M. stomatepiae*, in human disease.

We describe the first detailed case report of human infection with *M. stomatepiae.* This organism can now be added to the growing list of NTM that are opportunistic human pathogens, though it is likely to remain a very rare infective agent. Exposure probably occurs through water contact, or potentially through exposure to diseased fish. Like several other slow-growing mycobacteria, (e.g. *Mycobacterium haemophilum*, *Mycobacterium marinum* and *Mycobacterium ulcerans*) diagnosis by culture requires incubation at lower temperatures [3], and definitive identification is best performed by sequencing methods, including 16S rRNA gene sequencing. As NTM infections are an uncommon cause of lymphadenitis in children, clinical suspicion by both the treating clinician and the laboratory is important to flag potential cases, enabling appropriate laboratory set-up (including incubation of cultures at 30–32 and 35–37 °C) and reducing the risk of missed or delayed diagnosis.

## References

[1] Tortoli E (2014). Microbiological features and clinical relevance of new species of the genus *Mycobacterium*. Clin Microbiol Rev.

[2] van Ingen J, Totten SE, Heifets LB, Boeree MJ, Daley CL (2012). Drug susceptibility testing and pharmacokinetics question current treatment regimens in *Mycobacterium simiae* complex disease. Int J Antimicrob Agents.

[3] Pourahmad F, Cervellione F, Thompson KD, Taggart JB, Adams A (2008). *Mycobacterium stomatepiae* sp. nov., a slowly growing, non-chromogenic species isolated from fish. Int J Syst Evol Microbiol.

[4] Malama S, Munyeme M, Mwanza S, Muma JB (2014). Isolation and characterization of non tuberculous mycobacteria from humans and animals in Namwala District of Zambia. BMC Res Notes.

[5] Mandell GL, Bennett JE, Dolin R (2015). Mandell, Douglas, and Bennett's Principles and Practice of Infectious Diseases.

[6] Pourahmad F, Thompson KD, Adams A, Richards RH (2009). Detection and identification of aquatic mycobacteria in formalin-fixed, paraffin-embedded fish tissues. J Fish Dis.

